# Human Lung Microbiome on the Way to Cancer

**DOI:** 10.1155/2019/1394191

**Published:** 2019-07-29

**Authors:** Olga V. Kovaleva, Daniil Romashin, Irina B. Zborovskaya, Mikhail M. Davydov, Murat S. Shogenov, Alexei Gratchev

**Affiliations:** ^1^N.N. Blokhin National Medical Research Center of Oncology, Moscow, Russia; ^2^Faculty of Biology, Moscow State University, Moscow, Russia; ^3^I.M. Sechenov First Moscow State Medical University, Moscow, Russia; ^4^N.A. Lopatkin Institute of Urology, Moscow, Russia

## Abstract

Recent research on cancer-associated microbial communities led to the accumulation of data on the interplay between bacteria, immune and tumor cells, the pathways of bacterial induction of carcinogenesis, and its meaningfulness for medicine. Microbial communities that have any kind of impact on tumor progression and microorganisms associated with tumors have been defined as oncobiome. Over the last decades, a number of studies were dedicated to *Helicobacter pylori* and its role in the progression of stomach tumors, so this correlation can be regarded as proven. Involvement of bacteria in the induction of lung cancer has been largely ignored for a long time, though some correlations between this type of cancer and lung microbiome were established. Despite the fact that in the present the microbial impact on lung cancer progression has many confirmations, the underlying mechanisms are poorly understood. Microorganisms can contribute to tumor initiation and progression through production of bacteriotoxins and other proinflammatory factors. The purpose of this review is to organize the available data on lung cancer microbiome and its role in malignant tumor progression.

## 1. Introduction

A vast amount of highly diverse microorganisms inhabits the human organism. Microorganisms are present in all mucous membranes and participate in various physiological processes. The totality of microorganisms living in a human body (microbiome) appears to have a strong impact on human health. Recent studies demonstrate correlations between a particular composition of microbiome and a broad spectrum of diseases including autoimmune diseases, obesity, and even mental disorders [1].

Today much attention is focused on the investigation of human commensal microbiome. It is traditionally believed that human microbiome most strongly affects intestinal, skin, and mucous membranes. The smaller number of studies is dedicated to the investigation of lung microbiome, since the lung was supposed to be sterile for a long time due to the difficulties in cultivation of lung-specific microorganisms [2]. With the development of methods that do not require microorganism cultivation, a number of researches have demonstrated that there is a unique microbial community that inhabits the lungs [3]. PCR screening of bacterial 16S RNA showed that a much lower number of bacteria in comparison with upper airways inhabit the lungs and lower respiratory systems. Nevertheless, a lung mucous membrane has its own resident microbiome [4]. There are three existing key factors defining healthy lung microbiome: migration of microorganisms down from the upper airways, disposal of microorganisms by human organism, and local growth conditions [5].

## 2. Normal Lung Microbiome

Lung microbiome is a relatively new research area and remains to be poorly studied. Taking into account the high-throughput sequencing data analysis recently presented by separate research groups, it can be concluded that lung microbiome is phylogenetically diverse [6, 7]. According to several studies, there are two main phyla—*Bacteroidetes* and *Firmicutes*—that constitute lung microbiome [8, 9]. Some genera such as *Prevotella* and *Veillonella* prevail in a healthy lung [7]. In addition, the lower respiratory system is predominantly represented by genera *Pseudomonas*, *Streptococcus*, *Fusobacterium*, *Megasphaera*, and *Sphingomonas* [7, 10].

## 3. Lung Microbiome in Nononcology Disease

The relationship between microorganisms and various inflammatory lung diseases is a well-established issue. Tuberculosis remains to be one of the most significant diseases caused by bacteria—*Mycobacterium tuberculosis*. Tuberculosis is the leading cause of mortality among infectious diseases worldwide and is still a major challenge for the medicine. According to the WHO, the mortality of tuberculosis in 2015 was around 1.4 million. One of the reasons of such a high burden is a strong treatment resistance of *Mycobacterium tuberculosis* (MT) provided by a specific composition of its cell containing mycolic acid coverage and unique glycopeptidolipids—mycosides. Mycosides prevent MT cell from elimination by macrophages. Even after phagocytosis, MT cells are able to continue its vital functions inside macrophage's endosomes. Moreover, MT has a capacity to develop L-forms, which are significantly less virulent and often cause asymptomatic disease [11]. MT causes chronical inflammation of lung tissues related to phagocyte proliferation that leads to fibrosis development. Fibrosis can occur due to any type of inflammation independently of whether it was induced by infection or not. A number of histological studies demonstrate the correlation between lung cancer and fibrosis promoted by MT [12, 13]. The following bacteria are also associated with chronic lung inflammation though less frequently: *Haemophilus influenzae*, *Moraxella catarrhalis*, *Streptococcus pneumoniae*, *Haemophilus parainfluenzae*, *Staphylococcus aureus*, and *Pseudomonas aeruginosa* [14]. Constant persistence of these species turns the disease into a chronic form and chronic inflammation. During the inflammation, the microbial community of the lungs becomes unstable and its species composition changes frequently due to immune system activity. Such events result in a leakage of cell lysis products into a microenvironment increasing concentration of proteins, lipopolysaccharides (LPS), and peptidoglycans [15]. Pathogenic bacteria (i.e. *Haemophilus influenza*) often produce lipopolysaccharides (LPSs) that affect the immune system as a strong proinflammatory factor. Another example of microbiome involved in inflammatory lung pathology is COPD. Significant differences between lung microbiome of healthy and COPD patients were found using 16S RNA sequencing analysis. Notably, the presence of *Pseudomonas*, *Streptococcus*, *Prevotella*, and *Haemophilus* genera is mostly typical for patients with COPD [16]. Since chronic inflammation is now accepted as an important carcinogenic factor, the role of bacteria in the development of lung cancer attracts significant attention of researchers worldwide.

## 4. Lung Cancer Microbiome

In the past decade, numerous studies were published investigating microbiome in cancer patients. Some strong associations between different types of cancer and specific microorganisms were established [17] (Table 1).

Lung cancer is one of the most common types of cancer, and it is leading in the mortality rate among cancer patients. It can be promoted by a variety of factors including chemical carcinogens, chronic inflammation, bacterial and viral infections, periodontal diseases, and many others. Pathogenic and opportunistic pathogenic microorganisms are indeed capable to drive inflammation of lung tissues. This was demonstrated for such microorganisms like *Haemophilus influenzae*, *Enterobacter* spp., *E. coli*, *Pneumococcus* [27], *Legionella* [1], and *Moraxella* genera [21, 28]. Moreover, in some cases, these microorganisms are associated with lung cancer. There is also some specific association with a particular histologic type of tumor observed. For instance, genera *Acidovorax*, *Klebsiella*, *Rhodoferax*, *Comamonas*, and *Polarmonas* are more frequently found in small-cell carcinoma (SCC) and are not detected in adenocarcinoma cases [20].

Studies of infection-associated diseases including lung cancer have high priority for medicine. However, it should be noticed that sometimes lung cancer might be driven not by an infection itself, but by a significant shift in its microbial community. The diversity of lung microbiome is an important indicator of malignant transformation. Two types of diversity are distinguished—alpha and beta biodiversity. Alpha diversity (the number of species in one habitat) tends to be lower in lung cancer patients. Beta diversity (the diversity between habitats) in opposite does not differ significantly in healthy and cancer patient lungs [29].

The recent studies in this area confirm that microbiome should be considered an important diagnostic and preventive indicator. Lee and colleagues showed the difference between microbiomes of patients with benign and malignant tumors via high-throughput NGS sequencing of 16S rRNA. The authors suggested that genera *Veillonella* and *Megasphaera* may be potentially considered lung cancer biomarkers [30]. Greathouse with colleagues demonstrated a correlation between *Acidovorax* genus and small-cell carcinoma. The authors established that this genus is predominant for this histological type of tumor and is undetectable in adenocarcinoma cases. *Pseudomonas* genus shows a correlation with adenocarcinoma. A similar pattern can be seen in COPD patients [6].

Another research group revealed the involvement of *Granulicatella adiacens* in lung tumor development. In earlier works, the authors described the association between *Granulicatella adiacens* and other opportunistic pathogens—*Enterococcus* sp., *Streptococcus intermedius*, *Escherichia coli*, *Streptococcus viridans*, *Acinetobacter junii*, and *Streptococcus* sp. However, such correlation only can be observed in lung cancer cases and does not emerge in healthy patients. The authors also reported the correlation between the titer of *Granulicatella adiacens* and the disease status. Noteworthy, *Granulicatella adiacens* presence is more common in nonsmoker samples that in smoker samples [26].

*Capnocytophaga*, *Selenomonas*, *Veillonella*, and *Neisseria* genera can be highlighted inter alia of potential lung cancer biomarkers. Increasing titer of these microorganisms correlates with both small-cell carcinoma (SCC) and adenocarcinoma (AC). These results were obtained with 16S RNA sequencing of saliva samples of 30 patients (10 SCC, 10 AC, and 10 healthy donors) and confirmed with real-time PCR [31].

Other studies demonstrated that the presence of emphysema in lung cancer patients affects lung microbiome. Thus, prevailing *Firmicutes* (*Streptococcus*) and *Bacteroidetes* (*Prevotella*) can characterize microbial composition of cancer and emphysema patients rather than emphysema-only patients. *Proteobacteria* phylum (i.e., *Acinetobacter* and *Acidovorax*) is in contrast less commonly in lung cancer cases independently of emphysema presence. According to the authors, these results confirm the importance of lung microbiome analysis [32].

During the recent years, many works were dedicated to the investigation of microbiome and its role in anticancer immunotherapy efficiency testing. Kaderbhai and coauthors demonstrated antibiotic impact during non-small-cell carcinoma treatment with nivolumab and showed that antibiotics does not affect the therapy [33]. Another group demonstrated that resistance to checkpoint inhibition therapy may result from abnormal composition in microbial communities. Efficiency of this therapy decreased dramatically upon antibiotic use [34]. Identification of correlations between antibiotic therapy and immune status may drastically change the approach of antibiotic use in cancer patients. In a Lewis lung cancer murine model, it was established that therapy with ampicillin, vancomycin, neomycin sulfate, and metronidazole intensifies susceptibility to tumor progression. The authors suggest that commensal balanced microbiome contributes to antitumor response and cotreatment with probiotics may facilitate cisplatin growth inhibitory and proapoptotic effects [35].

At the present time, the literature provides contradictory data regarding the antibiotic effect on anticancer therapy, underlining the importance of further investigations in this area.

## 5. Microbiome and Lung Cancer: Underlying Mechanisms

**Figure 1:**
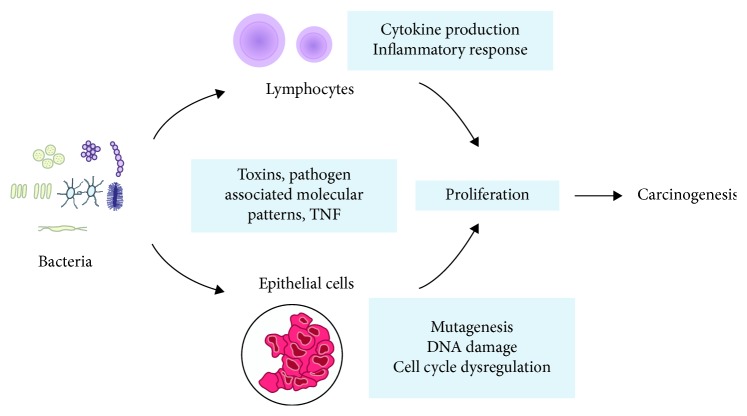
Interaction of microorganisms with epithelial cells and immune system cells, leading to carcinogenesis.

Mechanisms of potential bacterial impact on cancer initiation and progression are investigated for several decades now. These include direct effects via bacteriotoxins, inflammatory stimulation of immune cells, and direct effects on epithelial cells (Figure 1). Clearly, *Helicobacter pylori* is the best example of bacteria inducing gastritis, stomach ulcer, and cancer [36]. *Helicobacter pylori* toxin best known as CagA plays the key role in these processes. This toxin coded by the *CagA* gene interacts with epithelial cells facilitating bacterial cells to penetrate epithelium. Not all the *Helicobacter pylori* strains are capable of CagA synthesis. Thus, all strains are separated according to these criteria into CagA-positive and CagA-negative strains. It was reported that CagA-positive strains double the chances of stomach cancer in comparison to CagA-negative strains [22]. The specificity of carcinogenesis driven by *Helicobacter pylori* is remarkable. Ye et al. established that patients infected with CagA-positive *Helicobacter pylori* strains have a lower risk of esophagus adenocarcinoma than patients with CagA-negative strains [37].

A similar situation is described for colorectal carcinoma cases (CRC) where bacteriotoxin FadA promotes tumor development. FadA is a bacterial adhesin produced by *Fusobacterium nucleatum*. This protein binds E-cadherin and activates Wnt/*β*-catenin signaling which induces carcinogenesis [38]. *Fusobacterium nucleatum* is also capable of inhibiting apoptosis in tumor cells such as via Toll-like receptors and microRNA, which leads to tumor progression [39]. However, stomach cancer is not the only type of cancer related to *H. pylori*. A growing number of evidences suggest that *H. pylori* also induces oncology in the lungs [40]. Lipopolysaccharides of *H. pylori* may induce production of proinflammatory factors including IL-1, IL-6, and TNF. This inflammation may develop into chronic bronchitis that frequently accompanies lung cancer [41].

Commensal lung microbiome is crucial for immune homeostasis of a lung mucosal membrane. Disruptions in a lung microenvironment have an impact on susceptibility to several diseases including oncology. Cheng et al. demonstrated that mice exposed to oral antibiotic therapy had disruptions in *γδ*T17 T-cell functioning. Such disorders appear to increase receptivity to artificially induced B16/F10 melanoma and Lewis lung carcinoma (LLC). Meanwhile, antibiotic-resistant strains were not found and the total bacteria number decreased drastically. According to the authors, this work demonstrates that commensal microbiome is crucial for immune cell (*γδ*T17 cells) functioning [42].

Bacteriotoxins appear to play a significant role in tumor development. Cytolethal distending toxin (CDT), cytotoxic necrotizing factor 1, and *Bacteroides fragilis* toxin disrupt the DNA repair system which could lead to carcinogenesis [43–45]. Another in silico study showed that microcystin toxin of *Cyanobacteria* is related to decreasing of CD36 protein level and increasing concentration of PARP1 enzyme. Provided results were verified in a mouse model with NSCLC (A427) mice with bacteria-positive lung cancer [46]. Another research group established that TLR4 stimulation with heat-inactivated *E. coli* increases adhesion, migration, and metastatic spreading of non-small-cell lung cancer (NSCLC) cells *in vivo*. Such effects are particularly mediated by p38 MAPK and ERK1/2 signaling [47].

Apart from bacteriotoxins and direct influence of bacterial products, more general potentially carcinogenic mechanisms are known. Reactive oxygen species (ROS) is known to cause DNA damage. Recent studies demonstrate that shifts in microbiome composition may result in increasing ROS rates. Such event increases the DNA damage risk and predisposition to tumor development. It is important to mention that tumors carrying *TP53* mutations tend to associate with unique microbial communities. The latest studies indicate that mutations in *TP53* correlate with the presence of *Acidovorax* genus in the microenvironment. *Acidovorax* rates prevail in smokers' samples [20].

## 6. Conclusions

Lung microbiome investigations are a highly important challenge of modern biomedical science. It is well established that the lung contains specific microbial community regardless of population and geographic conditions. Lung microbiome is obviously correlated to a range of respiratory diseases. Certain spectrum of pathogenic microorganisms, in which amount and activity increases in the case of lung tumors, is already described, and new species are being added constantly. This provides a solid background for further investigation of lung cancer microbiome. Despite accumulating data, the mechanism of lung microbiome, immune system, and tumor interactions remains to be elusive. Understanding of this mechanism is indispensable of understanding the pathogenesis of lung cancer.

## Figures and Tables

**Table 1:** Associations between different cancer types and pathogenic microorganisms.

Organ	Microorganisms
Oral	*Fusobacterium nucleatum*, *Porphyromonas gingivalis* [17, 18]
Lung	*Haemophilus influenza*, *Acidovorax*, *Klebsiella*, *Moraxella catarrhalis*, *Mycobacterium tuberculosis*, *Granulicatella adiacens* [19–21]
Stomach, esophagus	*Helicobacter pylori* [22]
Pancreas	*Streptococcus mitis*, *Helicobacter pylori*, *Porphyromonas gingivalis* [18, 23]
Liver	*Helicobacter hepaticus* [24]
Intestine	*Escherichia coli*, *Fusobacterium nucleatum*, *Bacteroides fragilis*, *Enterococcus faecalis* [25, 26]
